# Estimating preferences and willingness to pay for pharmacogenetic testing in populations who are medically underserved: a discrete choice experiment

**DOI:** 10.3389/fphar.2024.1384667

**Published:** 2024-03-26

**Authors:** Brian E. Gawronski, Ramzi G. Salloum, Julio D. Duarte

**Affiliations:** ^1^ Department of Pharmacotherapy and Translational Research, College of Pharmacy, University of Florida, Gainesville, FL, United States; ^2^ Department of Health Outcomes and Biomedical Informatics, College of Medicine, University of Florida, Gainesville, FL, United States

**Keywords:** pharmacogenetic, medically underserved, discrete choice experiment, willingness to pay, implementation

## Abstract

**Background:** The implementation of pharmacogenetic (PGx) testing may contribute to health disparities if access to testing is inequitable, as medically underserved patients are prescribed higher rates of drugs with PGx guidelines and often experience the benefits of emerging health technologies last. Limited research has evaluated potential implementation of PGx testing in populations who are medically underserved and none have evaluated their preferences regarding PGx test characteristics and cost. Our study endeavored to assess the willingness to pay for PGx testing and key PGx test preferences in a nationwide cohort of medically underserved respondents.

**Methods:** A survey was developed to assess willingness to pay and preferences for PGx testing through a discrete choice experiment (DCE). Five attributes of PGx tests were included in the DCE: doctor recommendation, wait time, number of actionable results, benefit of the test (avoid a side effect or address a health problem), and out-of-pocket cost. A convenience sample of U.S. adults with an average yearly household income of $42,000 or less was collected utilizing an online survey fielded by Qualtrics Research Services (Provo, UT). For the DCE analysis, conditional logit and mixed-logit regression models were utilized to determine relative utility of attributes and levels, conditional relative importance for each attribute, and marginal willingness to pay.

**Results:** Respondents completed the survey with an 83.1% response completion rate. Following quality control procedures, 1,060 respondents were included in the final nationwide cohort. Approximately, 82% of respondents were willing to pay less than $100 for PGx testing, and a strong price ceiling was identified at $200. Out-of-pocket cost was the attribute identified as having the greatest relative importance on choice, while wait time had the lowest importance. Greater utility was observed if the PGx test was doctor recommended, had a higher number of actionable results, and resolved major or minor health problems compared with avoiding side effects.

**Conclusion:** This first-of-its-kind study provides important insights into the willingness to pay for PGx testing and PGx test preferences of a large medically underserved population. Applying these findings can potentially lead to improvements in the successful implementation of PGx testing in this population.

## 1 Introduction

Pharmacogenetic (PGx) testing, which tests for variants in genes involved in drug targets or in drug metabolism pathways, can be utilized to guide drug therapy providing meaningful therapy optimization for patients (Sadee, Wang et al., 2023). PGx testing has demonstrated clinical utility in decreasing adverse drug reactions (Swen, van der Wouden et al., 2023) and has shown promise in improving efficacy of medications (Pérez, Salavert et al., 2017). While PGx testing has been implemented into clinical practice, these experiences have been primarily limited to academic health centers (Dunnenberger, Crews et al., 2015), and for most, PGx testing remains an emerging health technology.

Emerging health technologies, such as PGx testing, have the potential to initially be available to those with higher socioeconomic status and only much later to medically underserved patients, who are often in greater need for the health technology. This phenomenon has been termed the inverse equity hypothesis (Victora, Vaughan et al., 2000). Medically underserved patients are usually the last patients to benefit from emerging health technologies (Weiss, Rydland et al., 2018), leading to the exacerbation of health inequities. The cost of PGx testing further limits access to testing for patients who are medically underserved. Further, our previous evaluation of the attitudes and perceptions within a medically underserved population found that the cost of testing was the largest concern with regards to PGx testing (Gawronski, Cicali et al., 2022). While information about PGx testing costs is scarce in the literature, a mean cost of $363.65 in 2014 United States Dollars (USD) was utilized in cost-effectiveness studies of PGx testing (Verbelen, Weale et al., 2017). This cost represents a significant barrier to PGx testing for populations who are medically underserved and may be without health insurance. Even in the context of health insurance, there is variability in the cost of PGx testing and a relatively low reimbursement rate (46%) (Lemke, Alam et al., 2023).

PGx testing may be at risk for following the inverse equity hypothesis, as we have previously reported that medically underserved patients are prescribed higher rates of drugs for which PGx guidelines are available (Dalton, Brown et al., 2021). Given the risk for the potential exacerbation of health inequities with PGx testing implementation, efforts to bolster implementation in groups experiencing health disparities should be undertaken (Victora, Joseph et al., 2018). Given the level of concern regarding the cost of testing, quantifying these groups’ willingness to pay for PGx testing is imperative to determine the barriers cost presents in these populations. While previous studies have evaluated the willingness to pay for PGx testing in various contexts and populations (Herbild, Gyrd-Hansen et al., 2008; Cuffe, Hon et al., 2014; Gibson, Hohmeier et al., 2017; Bereza, Coyle et al., 2020), it has yet to be evaluated in a predominantly medically underserved population.

While cost has been reported as the most significant concern with PGx testing in medically underserved populations, additional preferences may also drive the choice to undergo PGx testing. The discrete choice experiment (DCE) is a stated preference method which evaluates relative importance of aspects of a health intervention, service, or new technology (Ryan and Farrar, 2000). In DCEs, respondents, usually through a survey instrument, are given sets of hypothetical alternatives for which they are asked to choose their preferred alternative. DCEs have been utilized to evaluate preferences for PGx testing previously with success (Payne, Fargher et al., 2011; Dong, Ozdemir et al., 2016; Chen, Roberts et al., 2022), however no previous studies have evaluated the preferences of a medically underserved population. Therefore, we aimed to elucidate crucial PGx testing preferences and willingness to pay in a nationwide cohort of medically underserved respondents. This information can be utilized to guide successful implementations of PGx testing in these populations, avoiding the potential widening of disparities as this emerging health technology is implemented.

## 2 Materials and methods

### 2.1 Survey development

Details on the development and pilot testing of the survey instrument, which included questions on cost and willingness to pay, have been previously detailed (Gawronski, Cicali et al., 2022). A DCE, which was designed following the International Society for Pharmacoeconomics and Outcomes Research Good Research Practices for Conjoint Analysis Task Force (Bridges, Hauber et al., 2011; Reed Johnson, Lancsar et al., 2013), was included as a component of the survey instrument. An explanation of PGx testing as well as a section describing the DCE process and introducing the DCE attributes were included prior to the administration of 8 DCE choice tasks and 1 obvious choice test question.

The 5 attributes that were included (recommended by your doctor, wait (turnaround) time, number of actionable results in your lifetime, benefit of the test, and out-of-pocket cost) were chosen based on expert input as well as consultation of the literature. The determination of attribute levels was based on ensuring that clinically relevant options were included, while also including more extreme levels to ensure choice switching (the changing of a choice based on the given levels and attributes). Testing of attributes and attribute levels was completed by assessing responses of a pilot set of the first 100 respondents to the nationwide survey. All attributes and levels performed well and were included in the complete survey (Table 1). Whether the hypothetical test was recommended by a doctor was presented as a two-level attribute with “yes” or “no” as attribute levels. Wait (turnaround) time was presented with attribute levels of “0 days” or “3 days”. The number of times PGx test results would be actionable and used to help prescribe or adjust medications over the respondent’s lifetime included 1, 5, 10, and 15 as levels. PGx testing benefits were presented as either avoiding side effects or finding a medicine that was likely to work for a health problem in order to capture both potential benefits from PGx testing (Sadee, Wang et al., 2023). For avoiding side effects, “avoid a minor side effect” and “avoid a major side effect” were offered as attribute levels, and for finding a medicine that would solve a health problem, “find a medicine likely to work for a minor health problem” and “find a medicine likely to work for a major health problem” were offered as part of a choice set. Finally, out-of-pocket cost attribute levels included $0, $100, $200, and $300 USD. As part of the instructions for the DCE prior to the first choice set, a financial trade off reminder was included to emphasize the opportunity cost of spending money on the test during this part of the survey to limit the potential for hypothetical bias (Ozdemir, Johnson et al., 2009).

**TABLE 1:** Discrete choice experiment attributes and attribute levels.

Attributes	Attribute levels
Recommended by your doctor	Yes
	No
Wait (turnaround) time	0 days
	3 days
Number of actionable results in your lifetime	1
	5
	10
	15
Benefit of the test	Avoid a minor side effect
	Avoid a major side effect
	Find a medicine likely to work for a minor health problem
	Find a medicine likely to work for a major health problem
Out-of-pocket cost	$0
	$100
	$200
	$300

For each choice set, two hypothetical PGx test options were presented (Supplementary Figure S1). A fully text-based presentation was utilized given respondent preference and choice consistency compared to graphical presentations (Veldwijk, Lambooij et al., 2015). An opt-out option was not provided as an alternative in the choice sets given the potential for biased estimates with a neither option, as well as the impracticality of providing a *status quo* option given the attributes tested (Veldwijk, Lambooij et al., 2014; Campbell and Erdem, 2019; Determann, Gyrd-Hansen et al., 2019).

The experimental design was constructed utilizing the idefix package in R Statistical Software (v4.1.1; R Core Team 2021). The Coordinate Exchange Algorithm (CEA) function was utilized to design a Bayesian D-efficient optimal design (Traets, Sanchez et al., 2020). The design was 94.5% efficient with balance in levels within attributes. The optimal design included 16 choice sets for a saturated design. To ensure respondents were not overburdened by the number of choice sets, the choice tasks were blocked into two blocks of eight choice sets each. Respondents were randomly selected to respond to one of the two blocks. In addition to eight choice sets, an additional choice set in which one alternative was an obvious and unambiguously better choice was included to assess attention and understanding of the DCE (Supplementary Figure S1) (Lancsar and Louviere, 2008). The experimental design and choice sets were then coded into the Qualtrics Research Services (Qualtrics, Provo, UT) platform (Weber, 2021).

### 2.2 Survey sample

Full details on the survey sample have previously been reported (Gawronski, Cicali et al., 2022). Briefly, utilizing Qualtrics Research Services (Qualtrics, Provo, UT) and their propriety panels of survey respondents, a convenience sample was collected between 3/29/2022 and 04/19/2022. Inclusion criteria for the survey included age 18 years of age or older, residence within the United States, and average yearly household income of $42,000 USD or less, which represents 150% of the 2021 poverty threshold for a household of 4, where the average family household size in 2021 was 3.21 (United States Census Bureau, 2021; United States Census Bureau, 2022a). Qualtrics Research Services (Qualtrics, Provo, UT) offers survey respondents proprietary incentives/cash honorariums for survey participation. Survey responses were collected until 1,100 responses were collected with an oversampling to allow for the removal of low-quality responses. Quality control was conducted removing responses which included items such as speeding or flatlining of answers. Additionally, responses were removed if the obvious choice attention check question was not answered correctly (Abbey and Meloy, 2017). The study was approved by the University of Florida Institutional Review Board.

### 2.3 Statistical analysis

For perceived cost and willingness to pay data, summary statistics were derived. For the analysis of the DCE data, McFadden’s random utility model was utilized as the theoretical basis for the analysis of the choice data (McFadden, 1980). Random utility theory holds that people usually make choices between discrete alternatives based on maximizing utility, or benefit, however there is some randomness in decision making which stems from variations in valuation of utility across a sample. The DCE data analysis was conducted in accordance with ISPOR Conjoint Analysis Good Research Practices Task Force recommendations (Hauber, González et al., 2016). Regression analysis was conducted utilizing effects coding for the levels within an attribute (Bech and Gyrd-Hansen, 2005; Daly, Dekker et al., 2016). A conditional logit regression model was constructed utilizing the clogit function in the survival package. This model has been shown to be consistent with random utility theory and relates probability of a choice to the attribute characteristics defined by the levels (McFadden, 1973). To assess the assumption of scale heterogeneity (that all choice sets measure utility equally across all respondents and choice sets), which is central to conditional logit models, a random-parameters logit (or mixed-logit) regression model was constructed utilizing the mlogit package. These models provide coefficients which correspond to relative preference weights for the attribute levels in the model. To compare the relative importance of the attributes in the model, the conditional relative importance for each attribute was computed by taking the difference in the relative utility values between the most and least preferred levels in the attribute. Each difference was then scaled to a scale of 10 based on the attribute with the greatest calculated difference. Marginal willingness to pay (mWTP) for each level within an attribute was calculated by dividing the negative beta coefficient for each level by the beta coefficient for cost (Lancsar and Louviere, 2008).

Subgroup analysis was conducted on the willingness to pay measures and the DCE. Subgroups included self-identified race, ethnicity, highest level of education achieved, history of previous PGx testing, history of previous adverse drug reaction, interest level in PGx testing, health literacy level, and social deprivation index (SDI), which is a measure of neighborhood level socioeconomic characteristics (Butler, Petterson et al., 2013). To reduce dimensionality or due to small group sizes, willingness to pay was grouped as <$50, $50-$99, $100-$199, $200-$399, and $400 or more USD, race was grouped as Caucasian/White, African American/Black, and Other, and education was grouped as having a post-secondary education or not. For subgroup analysis of willingness to pay, Chi-squared and Fisher’s exact tests were utilized for categorical covariates and analysis of variance (ANOVA) was utilized with continuous covariates. For subgroup analysis of the DCE, SDI was split into quartiles. To test for differences in preferences between subgroups, models were constructed with interaction terms between each level of each attribute and the dummy coded covariate, thus modeling the differences in preferences between the subgroups. A Wald test, a joint test of significance, was utilized to compare all of the interaction terms across the model to determine if there was a difference between the subgroups. All analyses were performed utilizing R (v4.1.1; R Core Team 2021). *p*-values ≤0.05 were considered significant.

## 3 Results

### 3.1 Respondent characteristics

Of the 5,918 screened respondents, 1,656 respondents met inclusion criteria with 1,376 respondents completing the survey instrument, representing a 27.9% eligibility rate and 83.1% completion rate, respectively. Following quality control procedures, the final cohort consisted of 1,060 respondents. Full details regarding quality control as well as the numbers of respondents removed for each step have previously been reported (Gawronski, Cicali et al., 2022). Briefly, 188 respondents were removed through quality control procedures such as eliminating respondents who provided flatlined answers, such as selecting the first option for every question in the survey, and logically discordant answers. An additional 128 respondents were removed for incorrectly answering the obvious choice question. The respondents were 67% female with a median age of 42 years (Table 2). For self-reported race, 76.8% reported they were Caucasian/White, 13.6% Black/African American, and 3.5% Asian, and 11.7% of respondents reported they were Hispanic, Latino, or Latinx. Respondents lived in 48 of 50 States. Nearly half of respondents were on Medicaid or had no health insurance.

**TABLE 2:** Respondent demographics*.

Demographic characteristics		*n* = 1,060
Age, years (median (IQR))		42 (25)
Gender	Female	711 (67.1)
	Male	343 (32.4)
	Other	6 (0.6)
Self-Reported Race	Caucasian/White	814 (76.8)
	Black/African American	144 (13.6)
	Asian	37 (3.5)
	Another Race	25 (2.4)
	Mixed Race	23 (2.2)
	American Indian/Alaskan Native	16 (1.5)
	Pacific Islander/Native Hawaiian	1 (0.1)
Hispanic, Latino, Latinx	Yes	124 (11.7)
	No	931 (87.8)
	Did not know	5 (0.5)
Insurance	Medicaid	359 (33.9)
	Medicare	275 (25.9)
	Commercial	212 (20.0)
	No insurance	169 (15.9)
	Other government provided	45 (4.2)

*Demographics are summarized as count (%) unless otherwise specified; IQR: interquartile range.

### 3.2 Perceived pharmacogenetic test cost and willingness to pay

**FIGURE 1:**
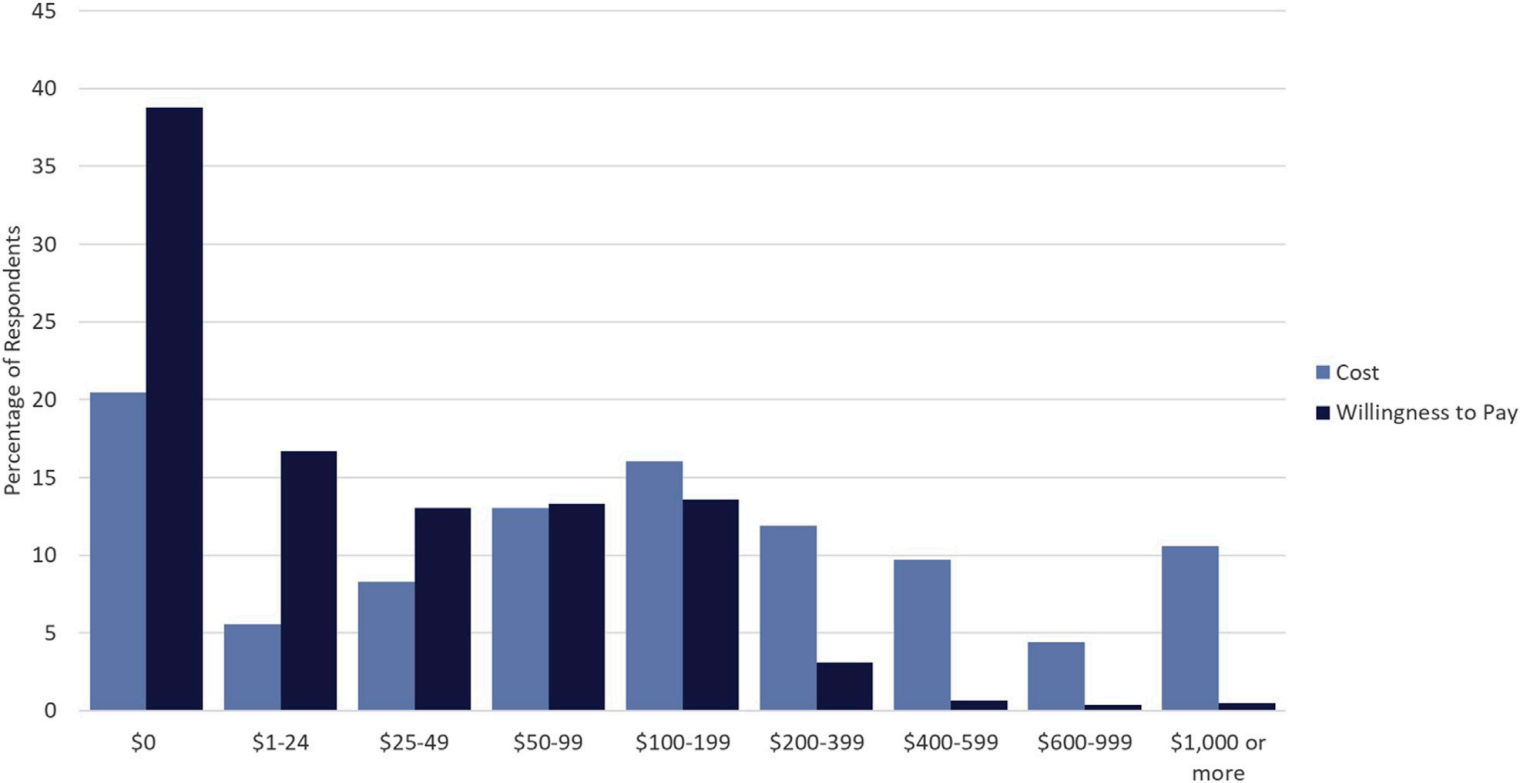
Cost *versus* Willingness to Pay for PGx Testing. Percentage of respondents who selected particular dollar amounts/ranges indicating perceived cost of PGx testing (light blue) and willingness to pay for PGx testing (dark blue).

Respondents were asked both what they think the current out of pocket cost for PGx testing is and what they would be willing to pay out of pocket for PGx testing. While respondents’ willingness to pay was skewed to the right with almost 39% willing to pay $0 USD for PGx testing, the perceived cost of testing was more normally distributed around $100–199 USD, with modes at both extremes (Figure 1). Approximately, 82% of respondents were willing to pay less than $100 USD for testing. Additionally, a sharp decline in the percentage of respondents willing to pay for PGx testing >$200 USD was observed, indicating that a strong price ceiling exists at costs above $200 USD.

### 3.3 Preferences regarding pharmacogenetic testing

**FIGURE 2:**
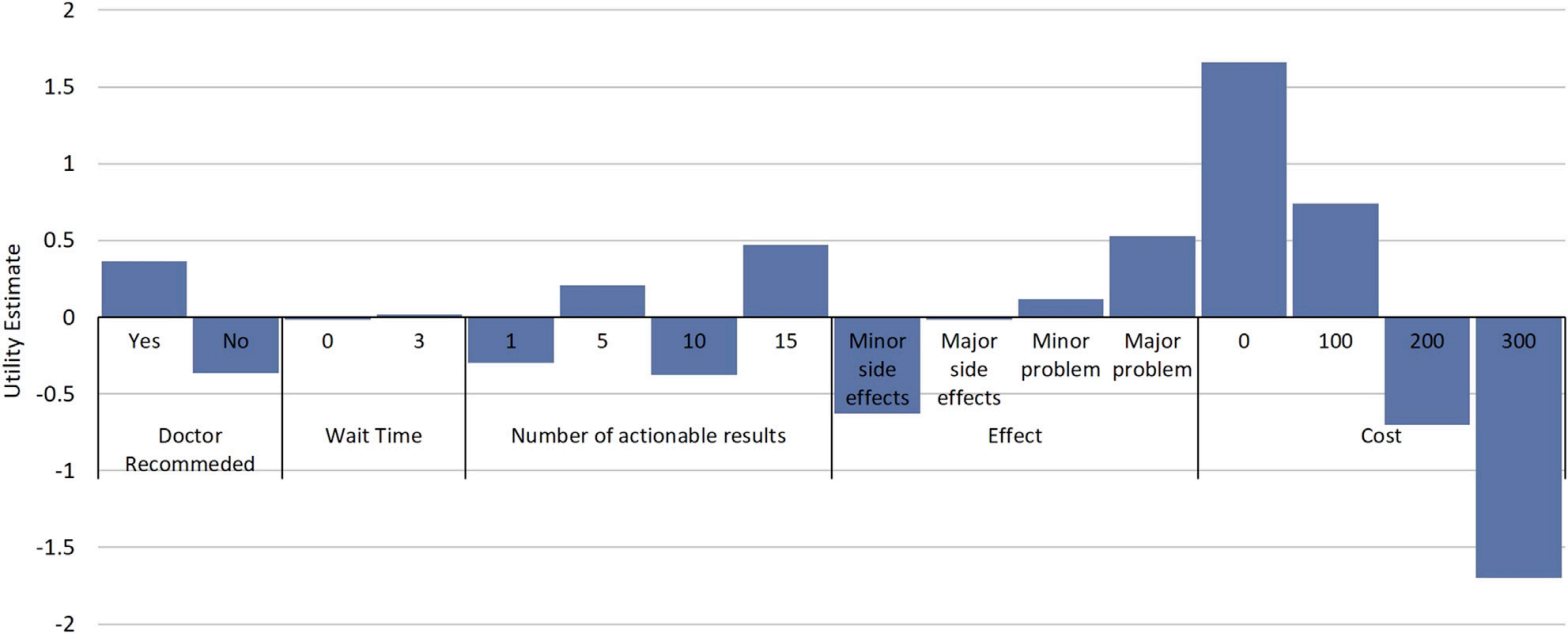
Relative Utility of Attributes and Attribute Levels. Estimated utilities for attribute and attribute levels from the discrete choice experiment. Estimates are from a mixed-logit model where data were effects coded. Utility estimates are relative between attribute levels of an attribute and comparisons cannot be made between attribute levels in different attributes.

**FIGURE 3:**
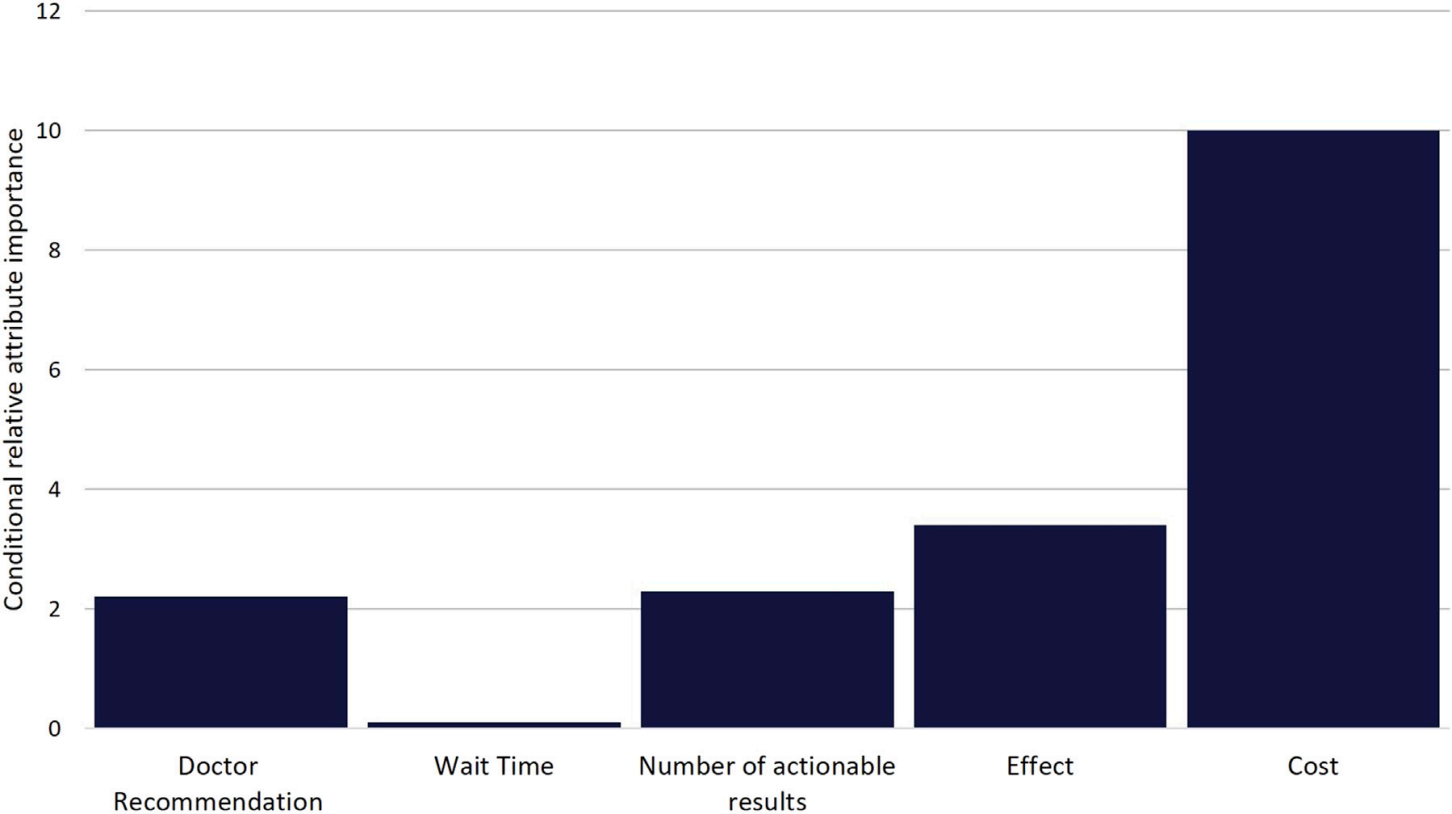
Scaled Conditional Relative Attribute Importance. Comparison of the relative importance of attributes on choice. Values were calculated based on the difference between the utility estimate of the attribute level with the highest positive utility and the utility estimate of the attribute level with the lowest negative utility for each attribute. Differences were then scaled based on the attribute with the greatest difference (Cost) being given a value of 10.

While DCE estimates and relative utility values results were similar between models, the mixed-logit regression model outperformed the conditional logit regression model (Table 3, Supplementary Table S1, Supplementary Figure S2). Considering the results of the mixed-logit model, the only attribute which did not show a statistically significant difference between levels was wait time, indicating the wait time for results between 0 and 3 days did not impact respondent choice (Table 4; Figure 2). Greater utility was observed if the PGx test was doctor recommended, had a higher number of actionable results, and resolved major or minor health problems compared with avoiding side effects. As cost increased, the utility of the PGx test decreased. When comparing the relative importance between attributes, changes in the level of out-of-pocket cost had the greatest conditional relative attribute importance with a value of 10 and thus influenced choice in PGx test the greatest relative to the other attributes. Out-of-pocket cost was followed by the effect of the testing with a value of 3.4, while wait time had the lowest importance with a value of 0.1 (Figure 3). This indicates the relative weighting of these attributes on PGx test preference.

**TABLE 3:** Comparison of regression model performance metrics*.

Model	AIC	Log-likelihood	R-squared
Conditional Logit	2,442,287	−1221,132	0.046
Mixed-Logit	8,028	−3,991	0.321

*AIC, akaike information criterion.

**TABLE 4:** Discrete choice experiment mixed-logit model relative utility estimates, significance level, and marginal willingness to pay (mWTP).

Attribute	Level	Estimate	*p*-Value	mWTP
Doctor Recommended	Yes	0.366	<2.2 × 10^−16^	$33.27
	No (ref)			
Wait Time	0 Days	−0.017	0.565	-$1.54
	3 Days (ref)			
Number of actionable results	15	0.470	8.1 × 10^−12^	$42.72
	10	−0.378	2.4 × 10^−7^	-$34.36
	5	0.208	2.9 × 10^−5^	$18.91
	1 (ref)			
Effect	Major problem	0.529	<2.2 × 10^−16^	$48.09
	Minor problem	0.115	0.014	$10.45
	Major side effects	−0.015	0.687	-$1.36
	Minor side effects (ref)			
Cost	$0	1.662	<2.2 × 10^−16^	-
	$100	0.742	<2.2 × 10^−16^	-
	$200	−0.704	<2.2 × 10^−16^	-
	$300 (ref)			

Considering the mixed-logit model results, respondents would be willing to pay approximately $48 USD more for a PGx test which solved a major health problem compared to a PGx test which prevented a minor side effect (Table 4). It is important to note that no significant difference was noted between the willingness to pay for a test which prevented a minor side effect and one that prevented a major side effect. Respondents would also be willing to pay $33.27 USD more for a test that was doctor recommended compared to one that was not doctor recommended.

### 3.4 Subgroup analysis

All subgroups, except ethnicity and history of previous adverse drug reaction had statistically significant differences in willingness to pay for PGx testing between groups (Supplementary Table S2). Respondents self-reporting as African American/Black, reporting post-secondary training/education, who had previously undergone PGx testing, and who had greater interest in PGx testing were willing to pay higher amounts for PGx testing compared to their comparator subgroups. Respondents with higher SDI scores, indicating higher levels of social deprivation, were willing to pay more (Supplementary Figures S3–S9). While all subgroups in the DCE had statistically significant differences in preferences for PGx testing (Supplementary Table S3), there were only small differences in relative utility between groups and similar results were seen in the subgroups when compared to the results for the entire sample (Supplementary Figures S10–S16).

## 4 Discussion

This study represents a first-of-its-kind study to assess the willingness to pay and PGx test preferences in a medically underserved population. To our knowledge this is also the first application of a DCE in respondents who are medically underserved in the field of Precision Medicine. Our findings indicate that the vast majority of respondents who were medically underserved were willing to pay less than $100 USD for PGx testing. Additionally, large pluralities of respondents were not willing to pay any price for PGx testing. These results provide quantification of out-of-pocket spending limits for medically underserved populations, and these limits put PGx testing out of reach of most of these respondents given current testing costs which average above $300 USD (Verbelen, Weale et al., 2017). Additionally, the apparent ceiling, over which very few indicated they would be willing to pay, appears to include out-of-pocket costs greater than $200 USD. However, due to hypothetical bias, hypothetical assessments of willingness to pay overestimate actual willingness to pay as much as 21% and thus the actual ceiling may be lower (Schmidt and Bijmolt, 2020). Thus, testing vendors/providers, insurance providers, and those implementing testing should strive for out-of-pocket testing costs below this ceiling.

Addressing PGx testing insurance coverage and out-of-pocket cost will be imperative for the successful implementation in medically underserved populations. On this front, recent Medicare coverage determinations (Centers for Medicare and Medicaid Services, 2021) may lead to insurance coverage expansion by other payors. However, implementation efforts in these populations will also require addressing subsidies for those who are uninsured. Additionally, our subgroup analysis identified subgroups (such as those who identify as Caucasian/White and those without post-secondary education/training) who were willing to pay less for PGx testing and would require monitoring for uptake in PGx testing implementations where testing is offered at an out-of-pocket cost that is generally acceptable to medically underserved populations.

Our study further identified key preferences for PGx tests for respondents and how much they value each of them. While cost was the most important factor, additional attributes were also deemed important. Respondents preferred PGx testing which solved health problems relative to tests that avoided adverse events. This important insight can guide marketing or outreach efforts to these patient populations, while also providing an opportunity for additional education on the impact of adverse events. Additionally, utility was noted for PGx tests which were doctor recommended, indicating that strong prescriber partnership will be essential for improving success of implementation in populations who are medically underserved. In general, the greater number of actionable results was associated with increased utility, however paradoxically 10 actionable results were associated with disutility. While our experimental design was balanced, we cannot determine if this result is a chance finding, the numbers 1 and 10 were viewed similarly by respondents in the survey instrument, or if there truly was not a perceived difference in utility between 1 and 10 actionable results. Finally, there was no significant difference between levels in the wait time for results attribute. While we only tested 0 and 3 days as wait time attribute levels, this result suggests that wait time could be extended as a trade-off to possibly reduce PGx testing cost or improve the feasibility of a PGx testing implementation. Additional study of the impact of longer wait times on PGx test choice is needed to further assess the potential to exchange the batching of samples (and its associated increased wait time) for lower costs.

This study is strengthened as both willingness to pay and a DCE were both conducted in a sample of respondents who would generally be considered medically underserved, an understudied population in the areas of precision medicine and PGx. Additionally, our study’s external validity and generalizability is strengthened by its sample size and reach. Our study represents the largest DCE evaluating PGx testing in any context to our knowledge, and the survey was completed by respondents across the United States. Additionally, robust subgroup analysis revealed that while there was statistically significant preference heterogeneity, relative utility measures in subgroups were consistent with those of the entire sample further strengthening our study.

There are certain limitations for the current study. While the demographics of our respondent sample are generally in line with those of the United States population (United States Census Bureau, 2022b), females were overrepresented, which is often observed in survey-based research (Becker, 2022), and respondents identifying as Hispanic, Latino, or Latinx were underrepresented, which may be related to the survey being only available in English. Additionally, convenience sampling conducted in this study could potentially allow for sampling bias. An additional limitation of the current study is the utilization of an income cut-off to identify respondents who are medically underserved. The use of geographic or other defining metrics such as primary care physicians *per capita*, while not feasible for this nationwide survey, may provide additional specificity in identifying respondents who are medically underserved. Income, however, has been utilized as a surrogate marker for medically underserved populations (Ricketts, Goldsmith et al., 2007), and income is well correlated with being medically underserved (Kim, Peterson et al., 2020). Additionally, as previously reported, our sample shares characteristics of a medically underserved sample and the median SDI for our sample is similar to that seen in other medically underserved populations (Patel, Krasnow et al., 2020; Gawronski, Cicali et al., 2022; Green, Larson et al., 2022). Despite these limitations and understanding local context is fundamental to successful implementation, these findings represent an important starting point to work from and further develop.

Additional limitations for this study are shared by all DCEs. A major limitation is that respondents’ true behavior, when actually having to expend money, may be different from the stated preferences in the study. While we did utilize cheap talk—or descriptions prior to the DCE which acknowledge the hypothetical nature of the choices, but ask respondents to make decisions as if actual money was going to be utilized to pay for the tests (Tonsor and Shupp, 2011)—to help mitigate this limitation, further research and additional study methods are necessary to completely assure external validity. An additional limitation is that attributes that could affect preferences for PGx tests may not have been included due to the need to balance including as many attributes as possible, while also ensuring respondents are also reasonably able to still make attribute informed choices. Limitations may also develop through attribute level selection when balancing a selection of levels which are different enough to influence choice, while not too extreme to dominate choice. This potential limitation can be most consequential for cost attribute levels (Ratcliffe, 2000; Rowen, Stevens et al., 2018), however we believe our selected attribute levels for cost are realistic and valid given the current costs of testing and the ability of our study population to pay. Finally, while we did evaluate multiple models to account for aspects of heterogeneity, we did not account for possible preference heterogeneity, or heterogeneity in preferences for certain subgroups within our studied sample. However, in the subgroup analysis we conducted while statistically significant differences in utility were noted, estimates of relative utility were not meaningfully different between the subgroups analyzed.

In conclusion, the results of this study provide important insights into the willingness to pay and preferences for PGx testing in populations who are medically underserved. Through the application of these findings, combined with the additional findings and validation through future research, improvements can be made in the successful implementation of PGx testing in populations who are medically underserved. The work remains to build upon the current study and apply the findings to early and proactive implementations of PGx testing in these populations and potentially prevent the widening of health disparities.

## Data Availability

The raw data supporting the conclusion of this article will be made available by the authors, without undue reservation.

## References

[B1] Abbey J. D. Meloy M. G. (2017). Attention by design: using attention checks to detect inattentive respondents and improve data quality. J. Operations Manag. 53-56 (1), 63–70. 10.1016/j.jom.2017.06.001

[B2] Bech M. Gyrd-Hansen D. (2005). Effects coding in discrete choice experiments. Health Econ. 14 (10), 1079–1083. 10.1002/hec.984 15852455

[B3] Becker R. (2022). Gender and survey participation: an event history analysis of the gender effects of survey participation in a probability-based multi-wave panel study with a sequential mixed-mode design. methods, data, anal. 16 (1).2022. 10.12758/mda.2021.08

[B4] Bereza B. G. Coyle D. So D. Y. Kadziola Z. Wells G. Grootendorst P. (2020). Stated preferences for attributes of a CYP2C19 pharmacogenetic test among the general population presented with a hypothetical acute coronary syndrome scenario. Clin. Outcomes Res. 12, 167–175. 10.2147/CEOR.S234298 PMC7090184 32256091

[B5] Bridges J. F. Hauber A. B. Marshall D. Lloyd A. Prosser L. A. Regier D. A. (2011). Conjoint analysis applications in health--a checklist: a report of the ISPOR Good research practices for conjoint analysis task Force. Value Health 14 (4), 403–413. 10.1016/j.jval.2010.11.013 21669364

[B6] Butler D. C. Petterson S. Phillips R. L. Bazemore A. W. (2013). Measures of social deprivation that predict health care access and need within a rational area of primary care service delivery. Health Serv. Res. 48 (2 Pt 1), 539–559. 10.1111/j.1475-6773.2012.01449.x 22816561 PMC3626349

[B7] Campbell D. Erdem S. (2019). Including opt-out options in discrete choice experiments: issues to consider. Patient 12 (1), 1–14. 10.1007/s40271-018-0324-6 30073482

[B8] Centers for Medicare and Medicaid Services (2021). Local coverage determination Pharmacogenomics testing L39073. Medicare coverage database. Available at: https://www.cms.gov/medicare-coverage-database/view/lcd.aspx?lcdid=39073#:∼:text=A%20Local%20Coverage%20Determination%20(LCD,jurisdiction%20that%20the%20MAC%20oversees (Accessed October 10, 2022). 26110197

[B9] Chen C. Roberts M. H. Raisch D. W. Thompson T. A. Bachyrycz A. Borrego M. E. (2022). Preferences for pharmacogenomic testing in polypharmacy patients: a discrete choice experiment. Per Med. 19 (6), 535–548. 10.2217/pme-2022-0056 36317592 PMC10859042

[B10] Cuffe S. Hon H. Qiu X. Tobros K. Wong C. K. A. De Souza B. (2014). Cancer patients acceptance, understanding, and willingness-to-pay for pharmacogenomic testing. Pharmacogenet Genomics 24 (7), 348–355. 10.1097/FPC.0000000000000061 24911662

[B11] Dalton R. Brown J. D. Duarte J. D. (2021). Patients with geographic barriers to health care access are prescribed a higher proportion of drugs with pharmacogenetic testing guidelines. Clin. Transl. Sci. 14 (5), 1841–1852. 10.1111/cts.13032 33955180 PMC8504817

[B12] Daly A. Dekker T. Hess S. (2016). Dummy coding vs effects coding for categorical variables: clarifications and extensions. J. Choice Model. 21, 36–41. 10.1016/j.jocm.2016.09.005

[B13] Determann D. Gyrd-Hansen D. de Wit G. A. de Bekker-Grob E. W. Steyerberg E. W. Lambooij M. S. (2019). Designing unforced choice experiments to inform health care decision making: implications of using opt-out, neither, or *status quo* alternatives in discrete choice experiments. Med. Decis. Mak. 39 (6), 681–692. 10.1177/0272989X19862275 31354031

[B14] Dong D. Ozdemir S. Mong Bee Y. Toh S. A. Bilger M. Finkelstein E. (2016). Measuring high-risk patients' preferences for pharmacogenetic testing to reduce severe adverse drug reaction: a discrete choice experiment. Value Health 19 (6), 767–775. 10.1016/j.jval.2016.03.1837 27712704

[B15] Dunnenberger H. M. Crews K. R. Hoffman J. M. Caudle K. E. Broeckel U. Howard S. C. (2015). Preemptive clinical pharmacogenetics implementation: current programs in five US medical centers. Annu. Rev. Pharmacol. Toxicol. 55, 89–106. 10.1146/annurev-pharmtox-010814-124835 25292429 PMC4607278

[B16] Gawronski B. E. Cicali E. J. McDonough C. W. Cottler L. B. Duarte J. D. (2022). Exploring perceptions, knowledge, and attitudes regarding pharmacogenetic testing in the medically underserved. Front. Genet. 13, 1085994. 10.3389/fgene.2022.1085994 36712853 PMC9880414

[B17] Gibson M. L. Hohmeier K. C. Smith C. T. (2017). Pharmacogenomics testing in a community pharmacy: patient perceptions and willingness-to-pay. Pharmacogenomics 18 (3), 227–233. 10.2217/pgs-2016-0161 28112585

[B18] Green B. B. Larson A. E. Huguet N. Angier H. Valenzuela S. Marino M. (2022). High blood pressure reduction, health insurance status, and social deprivation index in U.S. Community health centers. AJPM Focus 1 (2), 100018. 10.1016/j.focus.2022.100018 37791238 PMC10546572

[B19] Hauber A. B. González J. M. Groothuis-Oudshoorn C. G. M. Prior T. Marshall D. A. Cunningham C. (2016). Statistical methods for the analysis of discrete choice experiments: a report of the ISPOR conjoint analysis Good research practices task Force. Value Health 19 (4), 300–315. 10.1016/j.jval.2016.04.004 27325321

[B20] Herbild L. Gyrd-Hansen D. Bech M. (2008). Patient preferences for pharmacogenetic screening in depression. Int. J. Technol. Assess. Health Care 24 (1), 96–103. 10.1017/S0266462307080129 18218174

[B21] Kim S. J. Peterson C. E. Warnecke R. Barrett R. Glassgow A. E. (2020). The uneven distribution of medically underserved areas in chicago. Health Equity 4 (1), 556–564. 10.1089/heq.2020.0023 34095703 PMC8175250

[B22] Lancsar E. Louviere J. (2008). Conducting discrete choice experiments to inform healthcare decision making: a user's guide. Pharmacoeconomics 26 (8), 661–677. 10.2165/00019053-200826080-00004 18620460

[B23] Lemke L. K. Alam B. Williams R. Starostik P. Cavallari L. H. Cicali E. J. (2023). Reimbursement of pharmacogenetic tests at a tertiary academic medical center in the United States. Front. Pharmacol. 14, 1179364. 10.3389/fphar.2023.1179364 37645439 PMC10461057

[B24] McFadden D. (1973). “Conditional logit analysis of qualitative choice behaviour,” in Fontiers in econometrics. Editor Zarembka P. (New York, NY, USA: Academic Press), 105–142.New York

[B25] McFadden D. (1980). Econometric models for probabilistic choice among products. J. Bus. 53 (3), S13–S29. 10.1086/296093

[B26] Ozdemir S. Johnson F. R. Hauber A. B. (2009). Hypothetical bias, cheap talk, and stated willingness to pay for health care. J. Health Econ. 28 (4), 894–901. 10.1016/j.jhealeco.2009.04.004 19464743

[B27] Patel S. A. Krasnow M. Long K. Shirey T. Dickert N. Morris A. A. (2020). Excess 30-day heart failure readmissions and mortality in Black patients increases with neighborhood deprivation. Circ. Heart Fail 13 (12), e007947. 10.1161/CIRCHEARTFAILURE.120.007947 33161734 PMC8164383

[B28] Payne K. Fargher E. A. Roberts S. A. Tricker K. Elliott R. A. Ratcliffe J. (2011). Valuing pharmacogenetic testing services: a comparison of patients' and health care professionals' preferences. Value Health 14 (1), 121–134. 10.1016/j.jval.2010.10.007 21211494

[B29] Pérez V. Salavert A. Espadaler J. Tuson M. Saiz-Ruiz J. Sáez-Navarro C. (2017). Efficacy of prospective pharmacogenetic testing in the treatment of major depressive disorder: results of a randomized, double-blind clinical trial. BMC Psychiatry 17 (1), 250. 10.1186/s12888-017-1412-1 28705252 PMC5513031

[B30] Ratcliffe J. (2000). The use of conjoint analysis to elicit willingness-to-pay values. Proceed with caution? Int. J. Technol. Assess. Health Care 16 (1), 270–275. 10.1017/s0266462300161227 10815371

[B31] Reed Johnson F. Lancsar E. Marshall D. Kilambi V. Mühlbacher A. Regier D. A. (2013). Constructing experimental designs for discrete-choice experiments: report of the ISPOR conjoint analysis experimental design Good research practices task Force. Value Health 16 (1), 3–13. 10.1016/j.jval.2012.08.2223 23337210

[B32] Ricketts T. C. Goldsmith L. J. Holmes G. M. Randolph R. M. R. P. Lee R. Taylor D. H. (2007). Designating places and populations as medically underserved: a proposal for a new approach. J. Health Care Poor Underserved 18 (3), 567–589. 10.1353/hpu.2007.0065 17675714

[B33] Rowen D. Stevens K. Labeit A. Elliott J. Mulhern B. Carlton J. (2018). Using a discrete-choice experiment involving cost to value a classification system measuring the quality-of-life impact of self-management for diabetes. Value Health 21 (1), 69–77. 10.1016/j.jval.2017.06.016 29304943

[B34] Ryan M. Farrar S. (2000). Using conjoint analysis to elicit preferences for health care. BMJ 320 (7248), 1530–1533. 10.1136/bmj.320.7248.1530 10834905 PMC1118112

[B35] Sadee W. Wang D. Hartmann K. Toland A. E. (2023). Pharmacogenomics: driving personalized medicine. Pharmacol. Rev. 75 (4), 789–814. 10.1124/pharmrev.122.000810 36927888 PMC10289244

[B36] Schmidt J. Bijmolt T. H. A. (2020). Accurately measuring willingness to pay for consumer goods: a meta-analysis of the hypothetical bias. J. Acad. Mark. Sci. 48 (3), 499–518. 10.1007/s11747-019-00666-6

[B37] Swen J. J. van der Wouden C. H. Manson L. E. Abdullah-Koolmees H. Blagec K. Blagus T. (2023). A 12-gene pharmacogenetic panel to prevent adverse drug reactions: an open-label, multicentre, controlled, cluster-randomised crossover implementation study. Lancet 401 (10374), 347–356. 10.1016/S0140-6736(22)01841-4 36739136

[B38] Tonsor G. T. Shupp R. S. (2011). Cheap talk scripts and online choice experiments: "looking beyond the mean. Am. J. Agric. Econ. 93 (4), 1015–1031. 10.1093/ajae/aar036

[B39] Traets F. Sanchez D. G. Vandebroek M. (2020). Generating optimal designs for discrete choice experiments in R: the idefix package. J. Stat. Softw. 96 (3), 1–41. 10.18637/jss.v096.i03

[B40] United States Census Bureau (2021). America’s families and living arrangements: 2021. Available at: https://www.census.gov/data/tables/2021/demo/families/cps-2021.html (Accessed October 10, 2022).

[B41] United States Census Bureau (2022a). Poverty thresholds. Available at: https://www.census.gov/data/tables/time-series/demo/income-poverty/historical-poverty-thresholds.html (Accessed October 10, 2022).

[B42] United States Census Bureau (2022b). U.S. Census Bureau QuickFacts: United States. Available at: https://www.census.gov/quickfacts/fact/table/US/PST045222 (Accessed November 5, 2023).

[B43] Veldwijk J. Lambooij M. S. de Bekker-Grob E. W. Smit H. A. de Wit G. A. (2014). The effect of including an opt-out option in discrete choice experiments. PLoS One 9 (11), e111805. 10.1371/journal.pone.0111805 25365169 PMC4218820

[B44] Veldwijk J. Lambooij M. S. van Til J. A. Groothuis-Oudshoorn C. G. M. Smit H. A. de Wit G. A. (2015). Words or graphics to present a discrete choice experiment: does it matter? Patient Educ. Couns. 98 (11), 1376–1384. 10.1016/j.pec.2015.06.002 26117796

[B45] Verbelen M. Weale M. E. Lewis C. M. (2017). Cost-effectiveness of pharmacogenetic-guided treatment: are we there yet? Pharmacogenomics J. 17 (5), 395–402. 10.1038/tpj.2017.21 28607506 PMC5637230

[B46] Victora C. G. Joseph G. Silva I. C. M. Maia F. S. Vaughan J. P. Barros F. C. (2018). The inverse equity hypothesis: analyses of institutional deliveries in 286 national surveys. Am. J. Public Health 108 (4), 464–471. 10.2105/AJPH.2017.304277 29470118 PMC5844402

[B47] Victora C. G. Vaughan J. P. Barros F. C. Silva A. C. Tomasi E. (2000). Explaining trends in inequities: evidence from Brazilian child health studies. Lancet 356 (9235), 1093–1098. 10.1016/S0140-6736(00)02741-0 11009159

[B48] Weber S. (2021). A step-by-step procedure to implement discrete choice experiments in Qualtrics. Soc. Sci. Comput. Rev. 39 (5), 903–921. 10.1177/0894439319885317

[B49] Weiss D. Rydland H. T. Øversveen E. Jensen M. R. Solhaug S. Krokstad S. (2018). Innovative technologies and social inequalities in health: a scoping review of the literature. PLoS One 13 (4), e0195447. 10.1371/journal.pone.0195447 29614114 PMC5882163

