# Supplementary material for: Estimating preferences and willingness to pay for pharmacogenetic testing in populations who are medically underserved: a discrete choice experiment
Source: Front Pharmacol. 2024 Mar 26;15:1384667. doi: 10.3389/fphar.2024.1384667 (PMC11002086; doi:10.3389/fphar.2024.1384667)

# Supplementary Material

## Tables

### Supplementary Table S1. Comparison of conditional logit and mixed-logit regression DCE results.

|  |  | **Conditional Logit** | | | **Mixed-Logit** | | |
| --- | --- | --- | --- | --- | --- | --- | --- |
| **Attribute** | **Level** | **Estimate** | **P value** | **mWTP** | **Estimate** | **P value** | **mWTP** |
| Doctor Recommended | Yes | 0.451 | < 2x10^-16^ | $34.31 | 0.366 | < 2.2x10^-16^ | $33.27 |
|  | No (ref) |  |  |  |  |  |  |
| Wait Time | 0 Days | -0.012 | 0.0246 | -$1.42 | -0.017 | 0.565 | -$1.54 |
|  | 3 Days (ref) |  |  |  |  |  |  |
| Number of actionable results | 15 | 0.506 | < 2x10^-16^ | $32.17 | 0.470 | 8.1x10^-12^ | $42.72 |
|  | 10 | -0.372 | < 2x10^-16^ | -$20.32 | -0.378 | 2.4x10^-7^ | -$34.36 |
|  | 5 | 0.195 | < 2x10^-16^ | $10.65 | 0.208 | 2.9x10^-5^ | $18.91 |
|  | 1 (ref) |  |  |  |  |  |  |
| Effect | Major problem | 0.658 | < 2x10^-16^ | $50.95 | 0.529 | < 2.2x10^-16^ | $48.09 |
|  | Minor problem | 0.107 | 0.0662 | $4.77 | 0.115 | 0.014 | $10.45 |
|  | Major side effects | -0.014 | < 2x10^-16^ | -$2.55 | -0.015 | 0.687 | -$1.36 |
|  | Minor side effects (ref) |  |  |  |  |  |  |
| Cost | $0 | 1.916 | < 2x10^-16^ | - | 1.662 | < 2.2x10^-16^ | - |
|  | $100 | 0.825 | < 2x10^-16^ | - | 0.742 | < 2.2x10^-16^ | - |
|  | $200 | -0.841 | < 2x10^-16^ | - | -0.704 | < 2.2x10^-16^ | - |
|  | $300 (ref) |  |  |  |  |  |  |

DCE: discrete choice experiment; mWTP: marginal willingness to pay; ref: reference

### Supplementary Table S2. Willingness to pay for preemptive PGx test subgroup analysis statistical testing.

| Subgroup | Test | Test Statistic | P-value |
| --- | --- | --- | --- |
| Race | Fischer’s Exact Test | N/A | 0.002 |
| Ethnicity | Fischer’s Exact Test | N/A | 0.286 |
| Post-secondary education/training | Chi-Squared | 10.396 | 0.034 |
| SDI | ANOVA | 2.914 | 0.021 |
| Previous PGx testing | Fischer's Exact Test | N/A | <0.001 |
| History of adverse drug reaction | Chi-Squared | 1.095 | 0.895 |
| Interest in PGx testing | Fischer's Exact Test | N/A | <0.001 |

PGx: pharmacogenetic; SDI: Social Deprivation Index

### Supplementary Table S3. DCE subgroup analysis statistical test results.

| Subgroup | Wald Chi-square | p-value |
| --- | --- | --- |
| Race | 544.1 | <0.001 |
| Ethnicity | 400.5 | <0.001 |
| Post-secondary education/training | 868.0 | <0.001 |
| SDI | 410.6 | <0.001 |
| Previous PGx testing | 1054.7 | <0.001 |
| History of adverse drug reaction | 337.8 | <0.001 |
| Interest in PGx testing | 985.7 | <0.001 |

DCE: discrete choice experiment; PGx: pharmacogenetic; SDI: Social Deprivation Index

## Figures

### Supplementary Figure S1. Example choice set and obvious choice question. Example of a choice task question as it appeared in the survey instrument. Participants responded to 8 different choice tasks and one obvious choice task. This choice set was the obvious choice question which provided the extreme attribute levels for each test, where Test 1 was the obvious test choice. This question was utilized to assess understanding of the choice tasks and level of attention to the tasks.


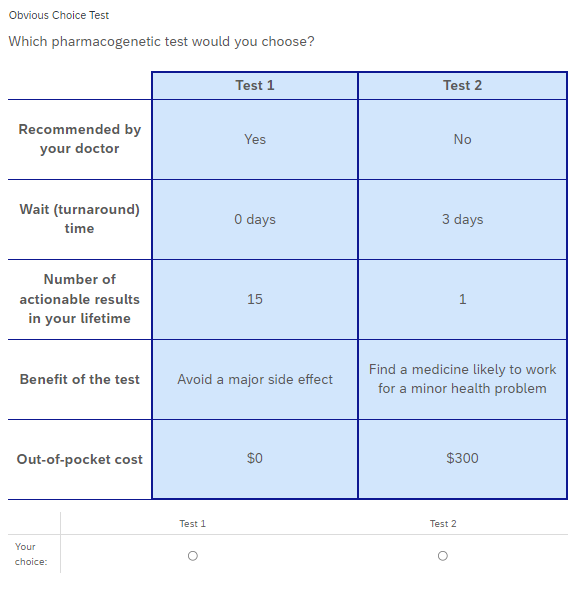


### Supplementary Figure S2. Comparison of relative utility between conditional logit and mixed-logit regression.


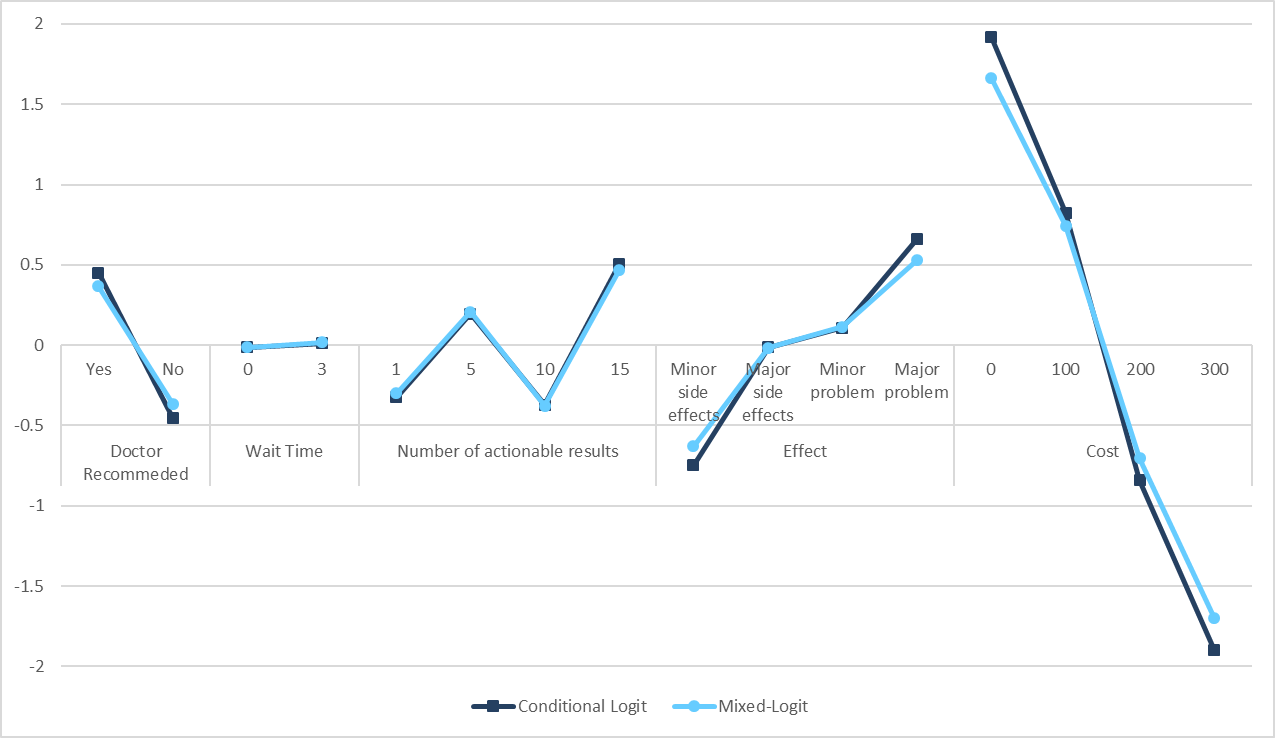


### Supplementary Figure S3. Willingness to pay for PGx testing subgroup analysis: race.


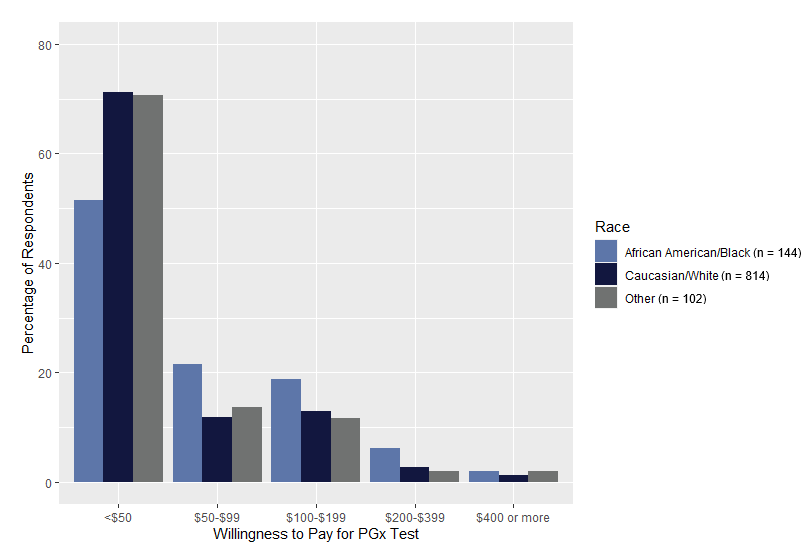


### Supplementary Figure S4. Willingness to pay for PGx testing subgroup analysis: ethnicity.


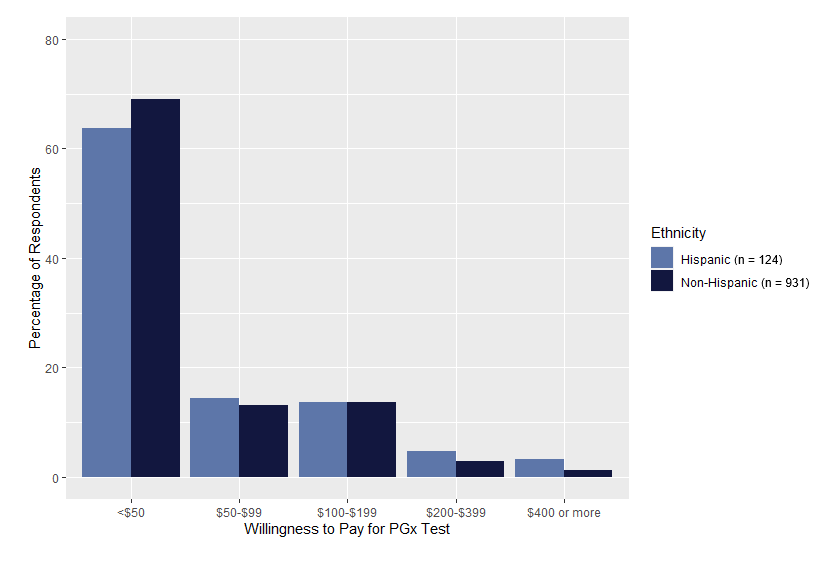


### Supplementary Figure S5. Willingness to pay for PGx testing subgroup analysis: post-secondary education/training.


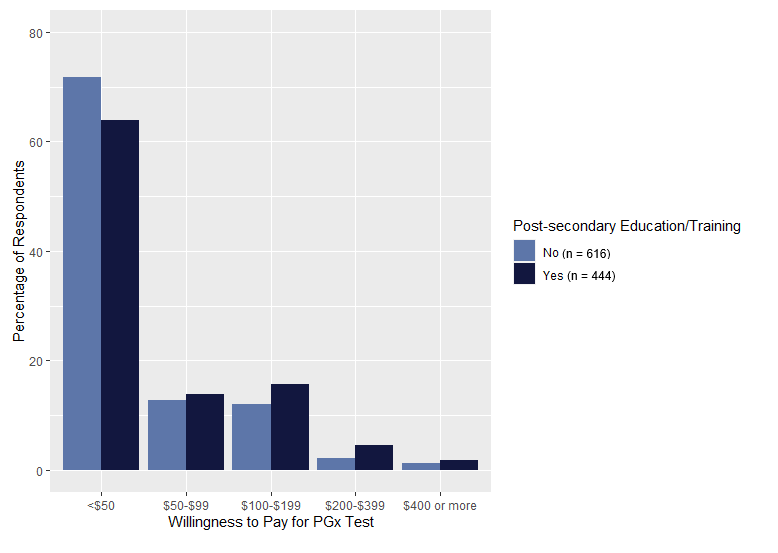


### Supplementary Figure S6. Willingness to pay for PGx testing subgroup analysis: social deprivation index (SDI). Dot plot of SDI by willingness to pay with horizontal lines indicating median.


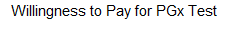

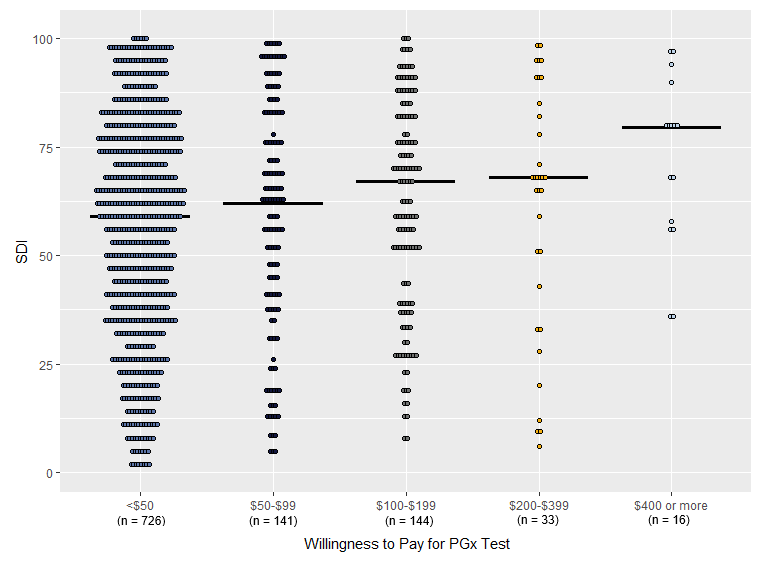


### Supplementary Figure S7. Willingness to pay for PGx testing subgroup analysis: previous PGx testing.


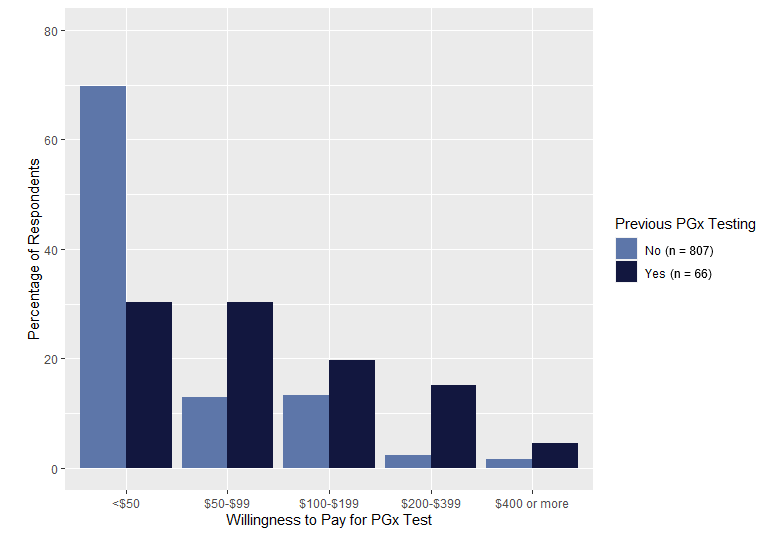


### Supplementary Figure S8. Willingness to pay for PGx testing subgroup analysis: history of adverse drug reaction.


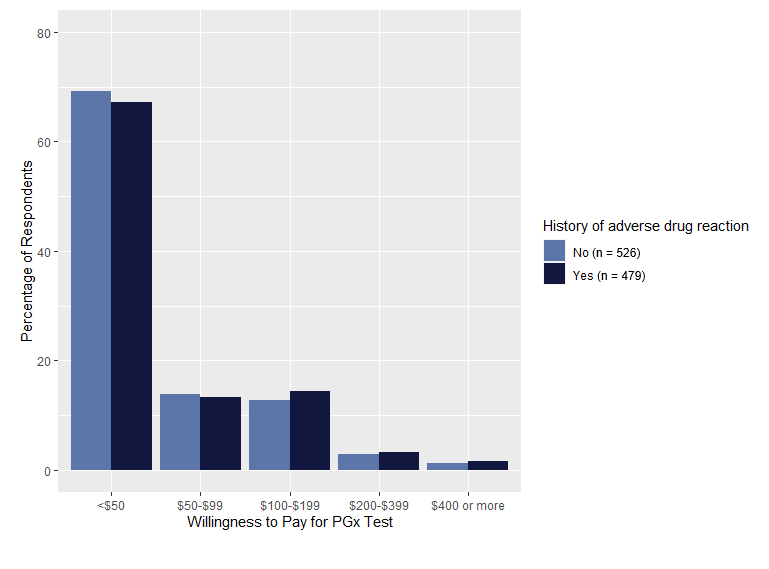


### Supplementary Figure S9. Willingness to pay for PGx testing subgroup analysis: interest in PGx testing.


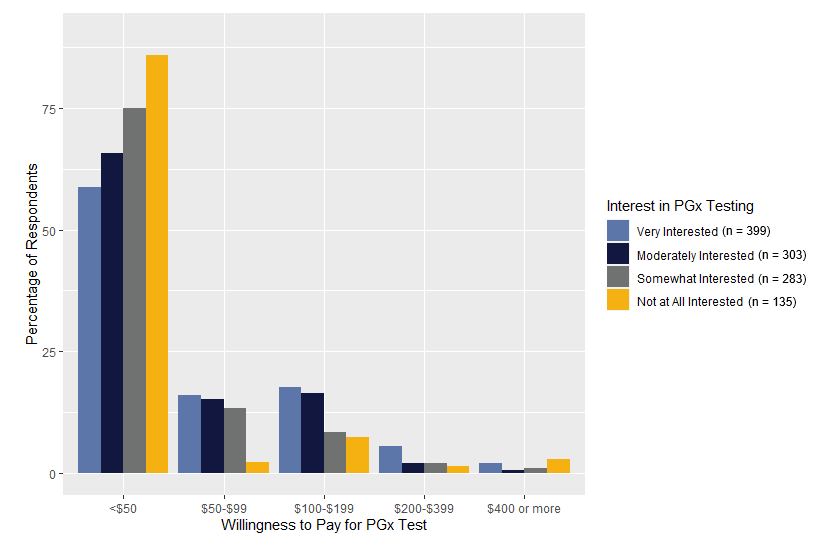


### Supplementary Figure S10. DCE subgroup analysis: race.


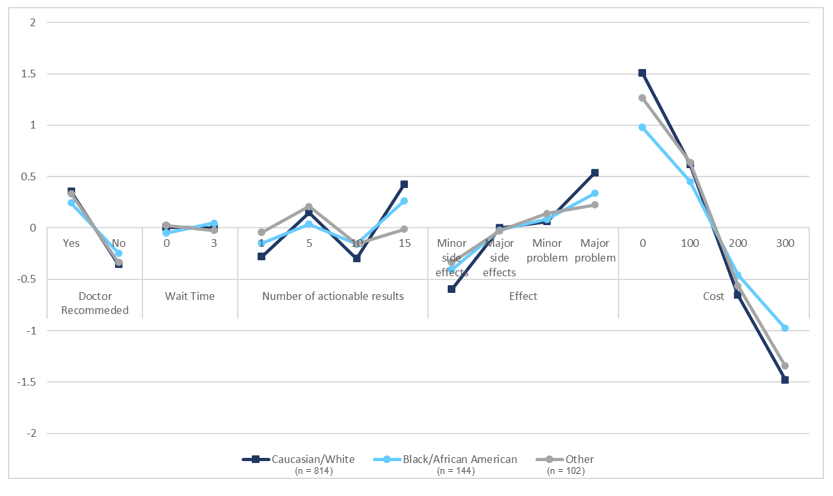


### Supplementary Figure S11. DCE subgroup analysis: ethnicity.


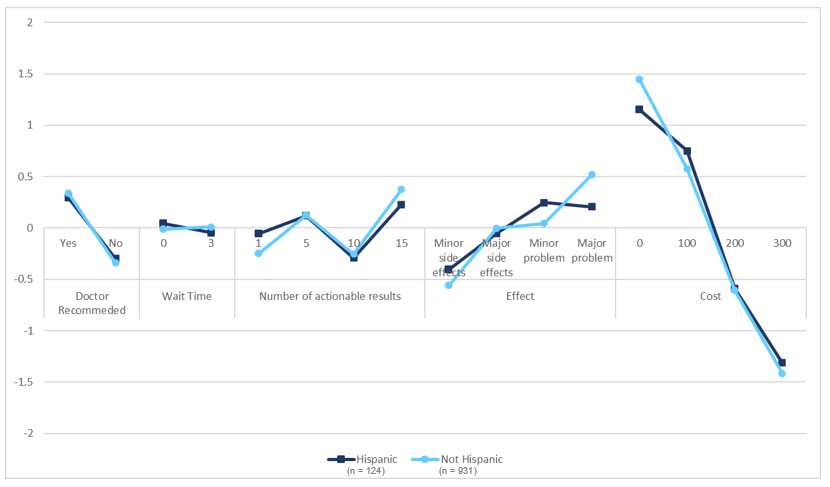


### Supplementary Figure S12. DCE subgroup analysis: post-secondary education/training.


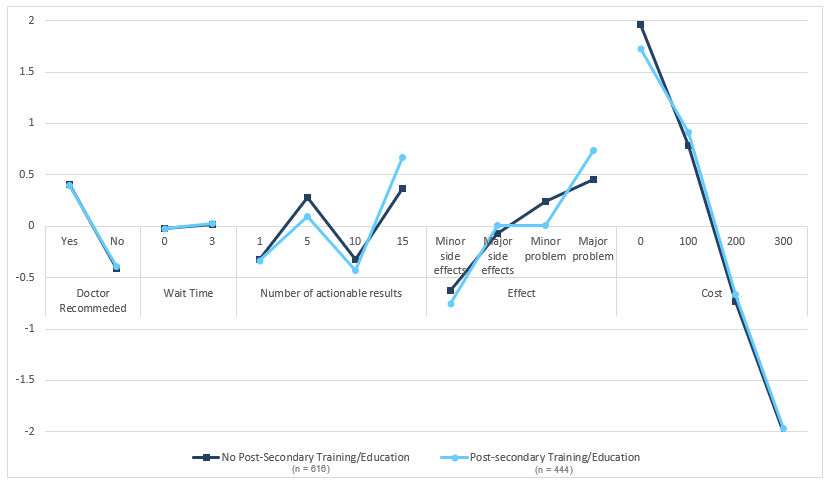


### Supplementary Figure S13. DCE subgroup analysis: social deprivation index (SDI).


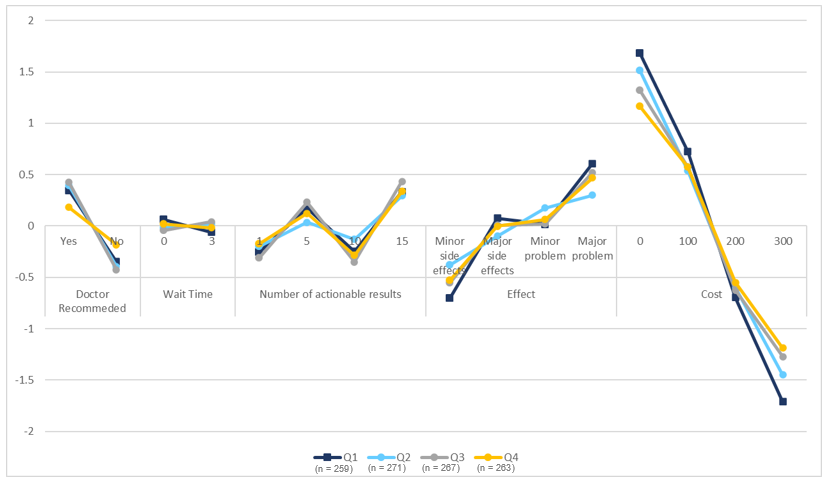


### Supplementary Figure S14. DCE subgroup analysis: previous PGx testing.


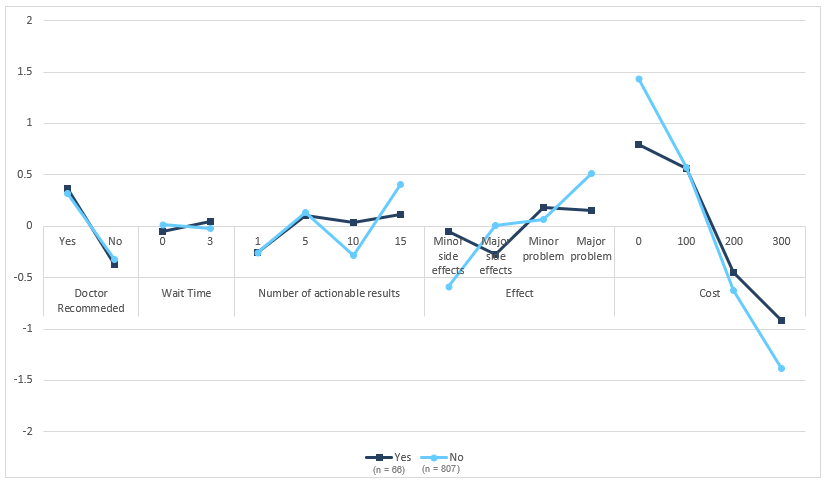


### Supplementary Figure S15. DCE subgroup analysis: history of adverse drug reaction.


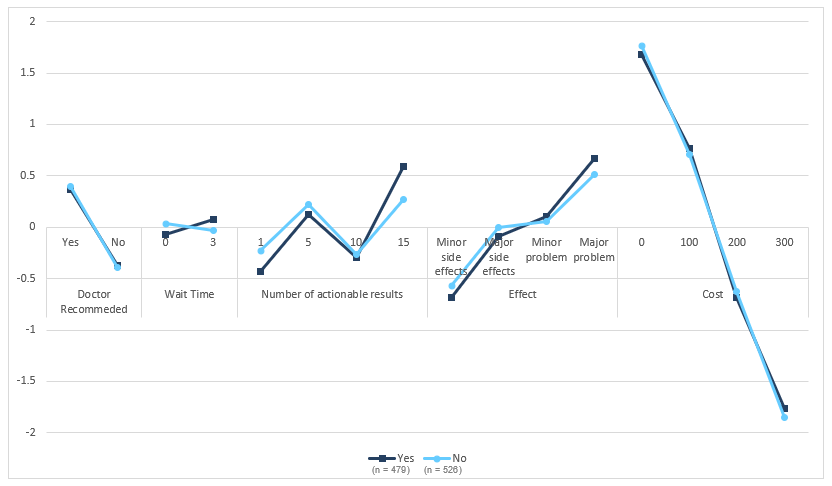


### Supplementary Figure S16. DCE subgroup analysis: interest in PGx testing.


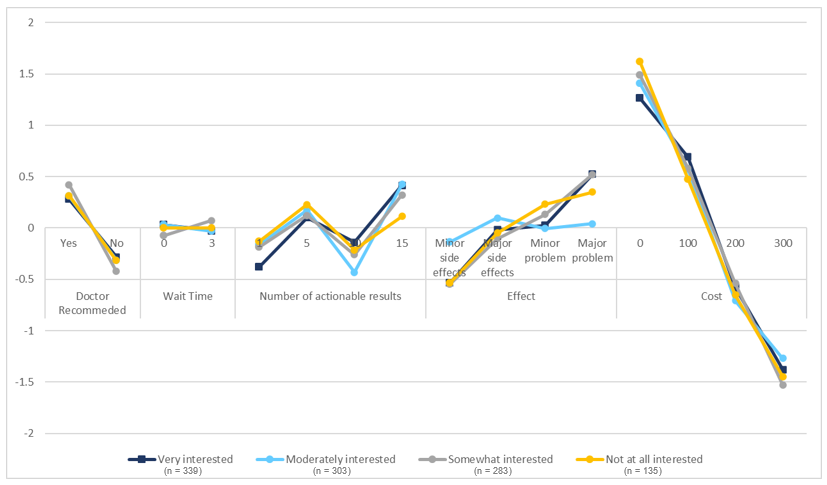

Supplement: Supplementary file 1 [file DataSheet1.docx]
